# Supplementary material for: Quantitative radiomics approach to assess acute radiation dermatitis in breast cancer patients
Source: PLoS One. 2023 Oct 26;18(10):e0293071. doi: 10.1371/journal.pone.0293071 (PMC10602246; doi:10.1371/journal.pone.0293071)
Supplement: S1 Checklist — (PDF) [file pone.0293071.s001.pdf]

PLOS ONE

PLOS ONE Clinical Studies Checklist

***PLOS ONE* manuscript number: ______PONE-D-23-06647______**

Complete the following if your study involved human participants or human subjects' data. These questions should be addressed for prospective and retrospective studies.

1. Did you obtain ethics approval for this study?

   - If yes, please upload (file type "Other") the original approval document you received from your ethics committee. If the original document is in another language, please also provide an English translation.

     _√_ _ Uploaded ___ N/A

   - If you did not obtain ethical approval, please explain why this was not required.

2. If your study involved human participants, please report in the Methods section when participants were recruited to the study.

   _√_ _ Completed ___ N/A

3. If you are reporting a study of medical records or archived samples, please report in the Methods section the date range in which human subjects' data/samples were collected and the date(s) when you conducted this study.

   _√_ _ Completed ___ N/A

4. Please specify in the Methods section whether authors had access to information that could identify individual participants during or after data collection.

   _√_ _ Completed ___ N/A

5. If you are reporting an observational study – i.e. cohort, case-control, and cross-sectional studies – we recommend that the work is reported as per the requirements of the STROBE guidelines, and that you provide a completed STROBE checklist as a Supporting Information file with your submission.

   The STROBE checklist was developed to improve the reporting of observational human subjects research, and is available here: http://strobe-statement.org/fileadmin/Strobe/uploads/checklists/STROBE_checklist_v4_combined_PlosMedicine.docx.

   _√_ _ Completed ___ N/A

6. Please ensure that the author list and Corresponding Author entered in Editorial Manager match the author list and Corresponding Author in your manuscript file.

   _√_ _ Completed
