# Supplementary material for: Quantitative radiomics approach to assess acute radiation dermatitis in breast cancer patients
Source: PLoS One. 2023 Oct 26;18(10):e0293071. doi: 10.1371/journal.pone.0293071 (PMC10602246; doi:10.1371/journal.pone.0293071)
Supplement: S1 Table — Four measurement points were placed on the upper, lower, inner, and outer sides of the ipsilateral breasts. (DOCX) [file pone.0293071.s002.docx]

**S1 Table.** Skin dose values measured using a nanoDot optically stimulated luminescent dosimeter (OSLD) for ipsilateral breasts. Four measurement points were placed on the upper, lower, inner, and outer sides of the ipsilateral breasts.

| **Patient ID** | **Upper (cGy)** | **Lower (cGy)** | **Inner (cGy)** | **Outer (cGy)** |
| --- | --- | --- | --- | --- |
| 1 | 260.2 | 251.7 | 221.8 | 266.3 |
| 2 | 271.4 | 245.5 | 244.0 | 233.8 |
| 3 | 256.0 | 247.4 | 202.8 | 284.0 |
| 4 | 272.2 | 229.5 | 238.1 | 199.3 |
| 5 | 262.2 | 231.0 | 240.5 | 213.1 |
| 6 | 265.2 | 237.7 | 212.6 | 253.7 |
| 7 | 262.9 | 232.1 | 208.4 | 223.1 |
| 8 | 228.5 | 224.7 | 186.7 | 219.8 |
| 9 | 264.6 | 227.9 | 209.5 | 236.6 |
| 10 | 247.4 | 217.3 | 209.7 | 204.9 |
| 11 | 249.3 | 275.0 | 200.9 | 217.6 |
| 12 | 240.4 | 243.0 | 203.5 | 248.9 |
| 13 | 264.0 | 274.6 | 261.8 | 250.9 |
| 14 | 246.1 | 273.7 | 214.4 | 202.1 |
| 15 | 255.6 | 265.1 | 199.7 | 223.9 |
| 16 | 232.9 | 244.2 | 204.4 | 212.4 |
| 17 | 234.0 | 246.0 | 207.2 | 213.7 |
| 18 | 223.1 | 273.1 | 189.1 | 203.5 |
| 19 | 267.9 | 263.4 | 198.4 | 226.4 |
| 20 | 259.0 | 252.7 | 193.7 | 201.9 |
| Mean | 253.1 ± 14.7 | 247.8 ± 18.1 | 212.4 ± 19.6 | 226.8 ± 23.5 |
