# Supplementary material for: Quantitative radiomics approach to assess acute radiation dermatitis in breast cancer patients
Source: PLoS One. 2023 Oct 26;18(10):e0293071. doi: 10.1371/journal.pone.0293071 (PMC10602246; doi:10.1371/journal.pone.0293071)
Supplement: S2 Table — (DOCX) [file pone.0293071.s003.docx]

**S2 Table.** *P*-values of a two-way repeated measures analysis of variance (ANOVA) in the three imaging modes

|  | Normal | |  | Polarised | |  | UV | |
| --- | --- | --- | --- | --- | --- | --- | --- | --- |
|  | Time | Time:group |  | Time | Time:group |  | Time | Time:group |
| Autocorrelation | 0.001 | 0.047 |  | < 0.001 | 0.012 |  | < 0.001 | < 0.001 |
| Contrast | < 0.001 | < 0.001 |  | < 0.001 | 0.025 |  | < 0.001 | 0.001 |
| Correlation | < 0.001 | 0.001 |  | - | - |  | - | - |
| Cluster prominence | < 0.001 | < 0.001 |  | - | - |  | - | - |
| Cluster shade | < 0.001 | 0.008 |  | - | - |  | - | - |
| Dissimilarity | < 0.001 | < 0.001 |  | < 0.001 | < 0.001 |  | < 0.001 | 0.003 |
| Energy | < 0.001 | < 0.001 |  | < 0.001 | 0.006 |  | - | - |
| Entropy | < 0.001 | < 0.001 |  | < 0.001 | 0.003 |  | < 0.001 | 0.007 |
| Homogeneity | < 0.001 | < 0.001 |  | < 0.001 | < 0.001 |  | < 0.001 | 0.001 |
| Maximum probability | < 0.001 | < 0.001 |  | - | - |  | - | - |
| Variance | 0.001 | 0.049 |  | < 0.001 | 0.012 |  | < 0.001 | < 0.001 |
| Sum average | < 0.001 | 0.042 |  | < 0.001 | 0.013 |  | < 0.001 | < 0.001 |
| Sum variance | 0.001 | 0.040 |  | < 0.001 | 0.010 |  | < 0.001 | < 0.001 |
| Sum entropy | < 0.001 | < 0.001 |  | < 0.001 | 0.009 |  | < 0.001 | 0.013 |
| Difference variance | < 0.001 | < 0.001 |  | < 0.001 | 0.025 |  | < 0.001 | 0.001 |
| Difference entropy | < 0.001 | < 0.001 |  | < 0.001 | < 0.001 |  | < 0.001 | 0.003 |
| IMC 1 | < 0.001 | 0.001 |  | < 0.001 | < 0.001 |  | < 0.001 | 0.011 |
| IMC 2 | < 0.001 | 0.002 |  | - | - |  | - | - |

*Abbreviations*: UV = ultraviolet; IMC = inverse measure of correlation.
