# Supplementary material for: Quantitative radiomics approach to assess acute radiation dermatitis in breast cancer patients
Source: PLoS One. 2023 Oct 26;18(10):e0293071. doi: 10.1371/journal.pone.0293071 (PMC10602246; doi:10.1371/journal.pone.0293071)
Supplement: S3 Table — (DOCX) [file pone.0293071.s004.docx]

**S3 Table.** *P*-values of a paired-t tests or Wilcoxon signed rank test in the three imaging modes.

|  | Normal | | |  | Polarised | | |  | UV | | |
| --- | --- | --- | --- | --- | --- | --- | --- | --- | --- | --- | --- |
|  | RT D7 | RT D14 | After RT D10 |  | RT D7 | RT D14 | After RT D10 |  | RT D7 | RT D14 | After RT D10 |
| Autocorrelation | - | - | - |  | 0.007 | < 0.001 | 0.015 |  | < 0.001 | < 0.001 | < 0.001 |
| Contrast | - | - | - |  | - | - | - |  | - | - | - |
| Correlation | - | - | - |  | - | - | - |  | - | - | - |
| Cluster prominence | - | - | - |  | - | - | - |  | - | - | - |
| Cluster shade | - | - | - |  | - | - | - |  | - | - | - |
| Dissimilarity | - | - | - |  | < 0.001 | < 0.001 | < 0.001 |  | - | - | - |
| Energy | 0.012 | < 0.001 | < 0.001 |  | - | - | - |  | - | - | - |
| Entropy | 0.006 | < 0.001 | 0.004 |  | 0.010 | 0.002 | 0.001 |  | - | - | - |
| Homogeneity | 0.007 | < 0.001 | 0.014 |  | < 0.001 | < 0.001 | < 0.001 |  | - | - | - |
| Maximum probability | - | - | - |  | - | - | - |  | - | - | - |
| Variance | - | - | - |  | 0.007 | < 0.001 | 0.015 |  | < 0.001 | < 0.001 | < 0.001 |
| Sum average | - | - | - |  | 0.007 | < 0.001 | 0.016 |  | < 0.001 | < 0.001 | < 0.001 |
| Sum variance | - | - | - |  | 0.006 | < 0.001 | 0.009 |  | < 0.001 | < 0.001 | < 0.001 |
| Sum entropy | 0.009 | < 0.001 | < 0.001 |  | - | - | - |  | - | - | - |
| Difference variance | - | - | - |  | - | - | - |  | - | - | - |
| Difference entropy | - | - | - |  | < 0.001 | < 0.001 | < 0.001 |  | - | - | - |
| IMC 1 | - | - | - |  | < 0.001 | < 0.001 | < 0.001 |  | - | - | - |
| IMC 2 | - | - | - |  | - | - | - |  | - | - | - |

*Abbreviations*: UV = ultraviolet; RT D*n* = approximately *n* days after the first day of the radiotherapy; After RT D*n* = approximately *n* days after the end of the radiotherapy; IMC = inverse measure of correlation.

Bonferroni's correction, which considered the adjusted significance level of 0.0167, was applied for *post hoc* comparisons.
