# Supplementary material for: Quantitative radiomics approach to assess acute radiation dermatitis in breast cancer patients
Source: PLoS One. 2023 Oct 26;18(10):e0293071. doi: 10.1371/journal.pone.0293071 (PMC10602246; doi:10.1371/journal.pone.0293071)
Supplement: Attachment — Submitted filename: Review report_comments_PLOS ONE.docx [file pone.0293071.s005.docx]

Hi

Overall this pilot study is conducted impressively from the point of view of clarity, objectivity, quantification, writing, presentation and new information.

However, I have few comments which needs response

1. Is skin dose measurement in this study using nano dot and OSLD is gold standard? And calibration

details?

2. Was the BMI taken into account (size the breast) and correlation with skin dose would be interesting?

3. Patient characteristics and Tumor characteristics does it have any effect on skin dose (eg. Age, color complexion (fair, brown and dark)?

4. Was the imaging dose( both CT and patient positioning verification dose)?

Thanks
