# Supplementary material for: Quantitative radiomics approach to assess acute radiation dermatitis in breast cancer patients
Source: PLoS One. 2023 Oct 26;18(10):e0293071. doi: 10.1371/journal.pone.0293071 (PMC10602246; doi:10.1371/journal.pone.0293071)
Supplement: Attachment — Submitted filename: Response_to_Reviewers.docx [file pone.0293071.s006.docx]

**Manuscript ID:** **PONE-D-23-06647**

**Title: Quantitative radiomics approach to assess acute radiation dermatitis in breast cancer patients**

We appreciate the reviewers’ time and effort in reviewing our manuscript. The quality of the manuscript has been substantially improved with the aid of the reviewer's valuable comments. The revised version of our manuscript has addressed the reviewer's concerns as detailed below.

**1. Journal requirements**

| **Journal requirements** | **Response** |
| --- | --- |
| 1. Please ensure that your manuscript meets PLOS ONE's style requirements, including those for file naming. | In accordance with the journal's requirements, the revised version of our manuscript was amended by referring to the PLOS ONE style templates. |
| 2. Please include this amended Role of Funder statement in your cover letter; we will change the online submission form on your behalf. | In accordance with the journal's requirements, we updated the funding information and added the role of the funder statement in our cover letter. |
| 3. Please remove any funding-related text from the manuscript and let us know how you would like to update your Funding Statement.  Please include your amended statements within your cover letter; we will change the online submission form on your behalf. | In accordance with the journal's requirements, we deleted any funding-related text from the revised version of our manuscript. |
| 4. We will update your Data Availability statement to reflect the information you provide in your cover letter. | In accordance with the journal's requirements, we updated the Data Availability statement in our cover letter. |
| 5. Please include your full ethics statement in the ‘Methods’ section of your manuscript file. In your statement, please include the full name of the IRB or ethics committee who approved or waived your study, as well as whether or not you obtained informed written or verbal consent. If consent was waived for your study, please include this information in your statement as well. | In accordance with the journal's requirements, we included our full ethics statement in the 'Methods' section of our revised manuscript, detailing the full name of the IRB, the informed written consent obtained, and the IRB number.  “This study was approved by the Institutional Review Board of Seoul National University hospital (IRB No. D-1810-053-977) and was conducted according to the Declaration of Helsinki. Informed written consent was obtained from all patients prior to their participation in this study.” |
| 6. Please review your reference list to ensure that it is complete and correct. | In accordance with the journal's requirements, we have reviewed and updated our reference list accordingly. |

**2. Reviewer #1**

| **Reviewer’s comment** | **Response** |
| --- | --- |
| Thank you for presenting to me the article “Quantitative radiomics approach to assess acute radiation dermatitis in breast cancer”. The idea of usings quantitative radiomics in the assessment of radiodermatitis is interesting. | We appreciate the comments from the reviewer. Comments from Reviewer #1 and the corresponding responses are listed below. |
| 1. The article lacks a clear research question and structure for answering this question. This is particular important since 1728 radiomic features per patient (line 181) are used. Moreover, one should be aware of the multiple comparison problem, e.g. finding significance by chance after doing lots of testing. | We appreciate the comments from Reviewer #1. As the reviewer pointed out, we should consider the multiple comparison problem when dealing with a considerable number of parameters. For statistical tests, we utilized the Benjamini-Hochberg correction to address multiple corrections and identify truly significant parameters. With this correction method, the adjusted p-values were calculated and are presented in Table 5. We have revised Table 5 and the corresponding sentences discussing this issue in both the 'Results' and 'Materials and Methods' sections.  “For these statistical tests, correction for multiple comparisons was performed with the Benjamin-Hochberg method to limit the false discovery rate at the 5% level. The adjusted *p*-values less than 0.05 were considered as statistically significant. All statistical analyses were performed using PASW statistic 18.0 software (SPSS, Chicago, IL, USA).” |
| 2. Rule 289: Why were the ipsilateral breasts used? | We appreciate the reviewer's comments. This study was conducted with a small sample of patients exhibiting specific conditions in the ipsilateral breast to enhance internal validation. In other words, our research, being a pilot study, evaluated whether the radiomics approach has the potential to objectively and efficiently assess the severity of radiodermatitis within a short period of time. In future work, we will expand on the concept of this study, incorporating various conditions and larger samples, to comprehensively assess skin damages caused by radiation using delta-radiomics. |
| 3. Practicality of the device? Experience? Errors? | We appreciate the reviewer's comments. Prior to beginning this study, we received training with the skin analysis device. We established an optimal protocol for skin measurement, conducting various simulations to minimize errors and uncertainty in this experiment. We can assure the integrity of the experimental protocol and device used in this study.  We have included sentences regarding the practicality, experience, and potential errors of the device in the 'Discussion' section.  “Before initiating the experiment, the skin analysis device utilized in this study was evaluated for variability and reproducibility of radiomic features under various conditions and environments. When measured more than five times in each of the three imaging modes under identical environmental conditions, the deviations in the radiomic feature values remained within 0.5%, demonstrating the device's good reproducibility. Even in extreme environmental scenarios, such as a dark room with approximately 100 lux, the device exhibited small deviations (< 0.5%) from radiomic feature values. The minimal effect of conditions and environments on this device can be attributed to its independent light source and the method of closely adhering to the patient's skin to acquire an image. By conducting various simulations, we established an optimal protocol for skin measurement to minimize errors and uncertainty in the experiment. Consequently, the bias in the correlation between skin damage and feature was reduced to the greatest extent possible.” |
| 4. What is the variability of radiomics when repeating the experiment at the same time, same patient and same location and same imaging mode? | We appreciate the reviewer's comments. We had already tested this skin analysis device for variability and responsiveness. We have included sentences regarding this issue in the 'Discussion' section.  “Before initiating the experiment, the skin analysis device utilized in this study was evaluated for variability and reproducibility of radiomic features under various conditions and environments. When measured more than five times in each of the three imaging modes under identical environmental conditions, the deviations in the radiomic feature values remained within 0.5%, demonstrating the device's good reproducibility. Even in extreme environmental scenarios, such as a dark room with approximately 100 lux, the device exhibited small deviations (< 0.5%) from radiomic feature values. The minimal effect of conditions and environments on this device can be attributed to its independent light source and the method of closely adhering to the patient's skin to acquire an image. By conducting various simulations, we established an optimal protocol for skin measurement to minimize errors and uncertainty in the experiment. Consequently, the bias in the correlation between skin damage and feature was reduced to the greatest extent possible.” |
| 5. What is the clinical benefit of using this device? | We appreciate the reviewer's comments. The clinical benefit of using this device is that it provides three different imaging modes. Among them, the polarized and UV modes can measure skin pigmentation and sebum, respectively, both originating from the basal layer of the epidermis. In other words, it allows for the evaluation of in-depth skin damages by considering not only the color and texture of the skin surface but also the deeper layers of the skin. |
| 6. Can this device assist radiotherapists/oncologists when assessing radiodermatitis? (without the use of radiomics) | We appreciate the reviewer's comments. The skin analysis device used in this study may assist radiotherapists and oncologists in providing quantitative measures for skin pore, pigmentation, and sebum. Among these, only skin pigmentation is known to be associated with radiation-induced skin damage. With such specific information, there are limitations in evaluating the severity of skin damage caused by radiation. For this reason, we applied radiomic analysis to obtain more comprehensive information. |

**3. Reviewer #2**

| **Reviewer’s comment** | **Response** |
| --- | --- |
| Overall this pilot study is conducted impressively from the point of view of clarity, objectivity, quantification, writing, presentation and new information.  However, I have few comments which needs response | We appreciate the comments from Reviewer #2. The comments and the corresponding responses are listed below. |
| 1. Is skin dose measurement in this study using nano dot and OSLD is gold standard? And calibration details? | We appreciate the reviewer's comments. We have included sentences regarding the detailed calibration protocol and the use of OSLDs as a measurement tool for skin doses in both the "Materials and Methods" and "Discussion" sections.  “For the calibration of pre-irradiated OSLDs, the absolute dose of linear accelerator was calibrated according to the American Association of Physicists in Medicine Task Group 51 protocol [38]. Subsequently, dose values ranging from 1 to 10 Gy were delivered to all sets of OSLD. The measurement setup consisted of a depth of maximum dose, an SSD of 100 cm, and a field size of 10 × 10 cm^2^.”  “The skin doses measured by OSLDs were reported as an absorbed dose at a skin depth of 0.4 mm, and this data was then utilized to assess the effect of radiation on the basal layer of the skin epidermis [49].” |
| 2. Was the BMI taken into account (size the breast) and correlation with skin dose would be interesting? | We appreciate the reviewer's comments. In this study, we prospectively selected Korean female patients with normal BMI and small breasts to enhance internal validation. As a feasibility study, we focused solely on examining the performance of radiomic analysis in evaluating radiodermatitis under specific, limited conditions. Future studies will be expanded to include a larger number of samples, various severities of radiation-induced skin damages, and participants from various races and multiple institutions. Additionally, skin doses will be measured using various dosimeters and calculated by radiation treatment planning systems.  “Therefore, further research using a large number of samples and various severities of radiodermatitis, various treatment sites, and various races from multiple institutions will be performed in the future. Skin doses will be measured by using various dosimeters and calculated by radiation treatment planning systems.” |
| 3. Patient characteristics and Tumor characteristics does it have any effect on skin dose (eg. Age, color complexion (fair, brown and dark)? | We appreciate the reviewer's comments. We were unable to find literature regarding the effect of patient characteristics and tumor characteristics on skin doses. However, we did find a study that reported the influence of race and ethnicity on the severity of radiation dermatitis. We have included sentences to discuss this issue in the revised manuscript.  “Additionally, it was reported that the expression of radiodermatitis was influenced by race and ethnicity, demonstrating that severe skin damage from radiation was more commonly observed in individuals with black skin than in those with white skin [50].” |
| 4. Was the imaging dose( both CT and patient positioning verification dose)? | We appreciate the reviewer's comments. In this study, we decided to focus solely on the treatment dose because the amount of imaging doses (0.2 ~ 2 cGy) from the CT scan and positioning verification system was about 100 times lower than the treatment dose (approximately 200 cGy). |

**4. Reviewer #3**

| **Reviewer’s comment** | **Response** |
| --- | --- |
| This is a well-carried out and well-written research on acute radiation dermatitis in patients undergoing breast cancer radiotherapy. It showed meticulous attention to details.  A pertinent question that may be addressed include: | We appreciate the comments from Reviewer #3. The comments and the corresponding responses are listed below. |
| 1. Is there a reason or reasons why scar boost is not given in these patients? Scar boost is standard in many breast conserving radiotherapy in many radiotherapy treatment guidelines. Could this impact future risk of disease recurrence on the irradiated breasts in these patients? | We appreciate the reviewer's comments. There is solid evidence that tumor bed boost after whole-breast radiotherapy further enhances local control in a patient with either invasive or in situ carcinoma. We deliver local tumor boost for all invasive cancer. For DCIS, the intuitional protocol was that tumor bed boost was to be delivered for lesions with involved resection margins or at the physician’s discretion. The NCCN guideline in 2023 endorsed both whole-breast RT only and shole-breast RT with tumor bed boost for DCIS. To eliminate confounding effect of boost on skin changes, we enrolled only patients with adequate margins for the current study and delivered no tumor bed boost in the study population. |
| 2. Detailed histopathological characterization of the patient's breast surgical specimens not provided, viz-a-viz resection margin status, DCIC with microinvasion [DCIS-M] etc. This could explain the lack of radiotherapy boost to tumor bed. | We appreciate the reviewer's comments. Surgical resection margins were clear and 2 mm or greater in all enrolled patients. |
